# Within-Site Variations in Soil Physicochemical Properties Explained the Spatiality and Cohabitation of Arbuscular Mycorrhizal Fungi in the Roots of *Cryptomeria Japonica*

**DOI:** 10.1007/s00248-024-02449-1

**Published:** 2024-11-04

**Authors:** Akotchiffor Kevin Geoffroy Djotan, Norihisa Matsushita, Kenji Fukuda

**Affiliations:** 1 Laboratory of Forest Botany, Graduate School of Agricultural and Life Sciences, University of Tokyo, Bunkyo, Japan; 2 Laboratory of Forest Mycology, Graduate School of Bioresources, Mie University, Tsu, Mie Japan

**Keywords:** Intraradical AMF community, Soil properties, Elevation, Community ecology, Japanese cedar

## Abstract

Arbuscular mycorrhizal fungi (AMF) live in a community in the roots of host plants. Still, the patterns and factors that drive their spatiality and cohabitation remain uncovered, particularly that of trees in planted forests, which we aimed to clarify in *Cryptomeria japonica*, a major plantation tree in Japan. We analyzed 65 paired root and soil samples of *Cryptomeria japonica* trees collected from 11 microsite (MS) plots at two environmentally different forest sites in central Japan and measured soil pH, total phosphorus (TP), C, N, and the carbon-to-nitrogen ratio. Root AMF communities were recovered using Illumina’s next-generation amplicon sequencing targeting the small subunit of ribosomal DNA. We detected more than 500 AMF OTUs at each site but only three belonging to *Dominikia*, *Rhizophagus*, and *Sclerocystis* were dominant in the roots of *C. japonica*, detected each at an average relative abundance higher than 20%. Two showed negatively correlated spatial distributions and different associations with soil pH. Similarly, the physicochemical properties at MSs significantly determined the AMF assemblages in the roots of *C. japonica*. *Dominikia*, *Rhizophagus*, and *Sclerocystis* coexist in the roots of *C. japonica* where soil physicochemical properties, particularly pH, determine their spatial dynamic, turnovers, and cohabitation patterns. These findings highlight the importance of simultaneous colonization of plants by multiple AMF.

**Supplementary Information:**

The online version contains supplementary material available at 10.1007/s00248-024-02449-1.

## Introduction

Soil microbial communities support the functions of plant communities through complex plant–microbe interactions [1]. In forest ecosystems, variations in abiotic soil conditions at scales from sub-millimeter to hundreds of meters, which are heterogeneous environmental conditions at those scales [2, 3], lead to fluctuations in the composition of soil microbial communities [4]. These scale-dependent effects of environmental forces also apply to arbuscular mycorrhizal fungi (AMF) [5]. AMF, which are omnipresent, asexual, and obligately symbiotic fungi, occupy a double habitat, comprising soil and the roots of vascular plants [6, 7]. These two habitats are exploited in differing manners by various AMF taxa [8–11], and the effects of soil physicochemical properties on the AMF community may differ between root and soil habitats [8, 12].

AMF community composition is also influenced by stochastic processes but responds predictably to the major abiotic niche axis of soil pH [13]. In addition to soil pH, other commonly discussed soil physicochemical properties that affect the AMF community include soil moisture and the concentrations of carbon (C), nitrogen (N), phosphorus (P), salts, and other mobile ions [14–20]. Furthermore, soil disturbance caused by human activities or erosion has been reported to affect the AMF community [21, 22]. These soil physicochemical properties may vary greatly within sites at fine spatial scales, affecting the composition of local AMF communities [2, 4, 23]. To account for these previous facts, we hypothesized that within-site variations in soil physicochemical properties induce changes in the composition of root AMF community spatially and affect the cohabitation of dominant AMF. To the best of our knowledge, such hypothesis has not been tested, particularly in forest tree species.

Spatial variations in AMF communities have been studied at fine-to-global scales [14, 23–30]. In most such studies, the biogeography of AMF and the factors that affect their spatial turnover in roots and soils were investigated. However, in the currently available literature, the spatial distribution of AMF has been less frequently investigated in the roots of trees than in forest soils. In addition, most related studies have covered numerous host species at multiple sites via soil coring, making it difficult to exclude the effect of host species in the analysis of the spatial covariation of root AMF communities and soil properties. Thus, the within-site spatial covariation of the root AMF community associated with a specific tree species and soil physicochemical properties of a forest ecosystem has not yet been clarified through high-throughput sequencing.

Plantations of *Cryptomeria japonica* (Cupressaceae) in Japan cover areas within the country where the natural landscape features mountains and valleys. Knowledge of the factors that affect the composition of the root AMF community of *C. japonica* at various spatial scales is limited. Soil conditions such as soil organic matter content, electrical conductivity, potassium, pH, and P and N concentrations affect the root and soil AMF communities in planted forests of *C. japonica* [8, 9, 31, 32]. However, the within-site spatial variations in soil physicochemical properties and their effects on the root AMF community of *C. japonica* remain to be investigated.

We aimed to elucidate the associations between soil physicochemical properties and root AMF communities at local scales within sites along topographic profiles. For this purpose, we set up microsite plots (MS) along topographic profiles of two planted forests of *C. japonica* located in environmentally differing sites, from which we collected pairs of root and soil samples; measured soil pH, total phosphorus (TP), total carbon (TC), total nitrogen (TN), and the carbon-to-nitrogen ratio (C/N); and characterized root AMF communities via Illumina MiSeq next-generation sequencing targeting the small subunit ribosomal DNA (SSU rDNA). Then, we analyzed the covariation between the measured soil physicochemical properties and the root AMF community.

## Material and Methods

### Study Sites and Sampling Design

In August 2021, we collected paired samples of roots of *C. japonica* trees and surrounding soils at the University of Tokyo Chiba Forest (UTCBF) and University of Tokyo Chichibu Forest (UTCF). The mean annual temperature and mean annual precipitation at UTCBF/UTCF are 14.0 °C/11.2 °C and 2500 mm/1498 mm, respectively [8].

The investigated plantations at UTCBF and UTCF were established in 1931 and 1980–1987, respectively, on landscapes with steep slopes. The stand density at the time of sampling was 750 and 1050 trees/ha at UTCBF and UTCF, respectively. The understory of the plantation at UTCBF was covered with abundant shrubs and herbaceous plants (current study) while the understory at UTCF contained few plants [8]. The understory plant communities were uniform at both sites and constituted of AMF hosts. The diameters at breast height of *C. japonica* trees were 56.7 ± 10.0 and 28.28 ± 4.1 cm at UTCBF and UTCF, respectively.

**Fig. 1:**
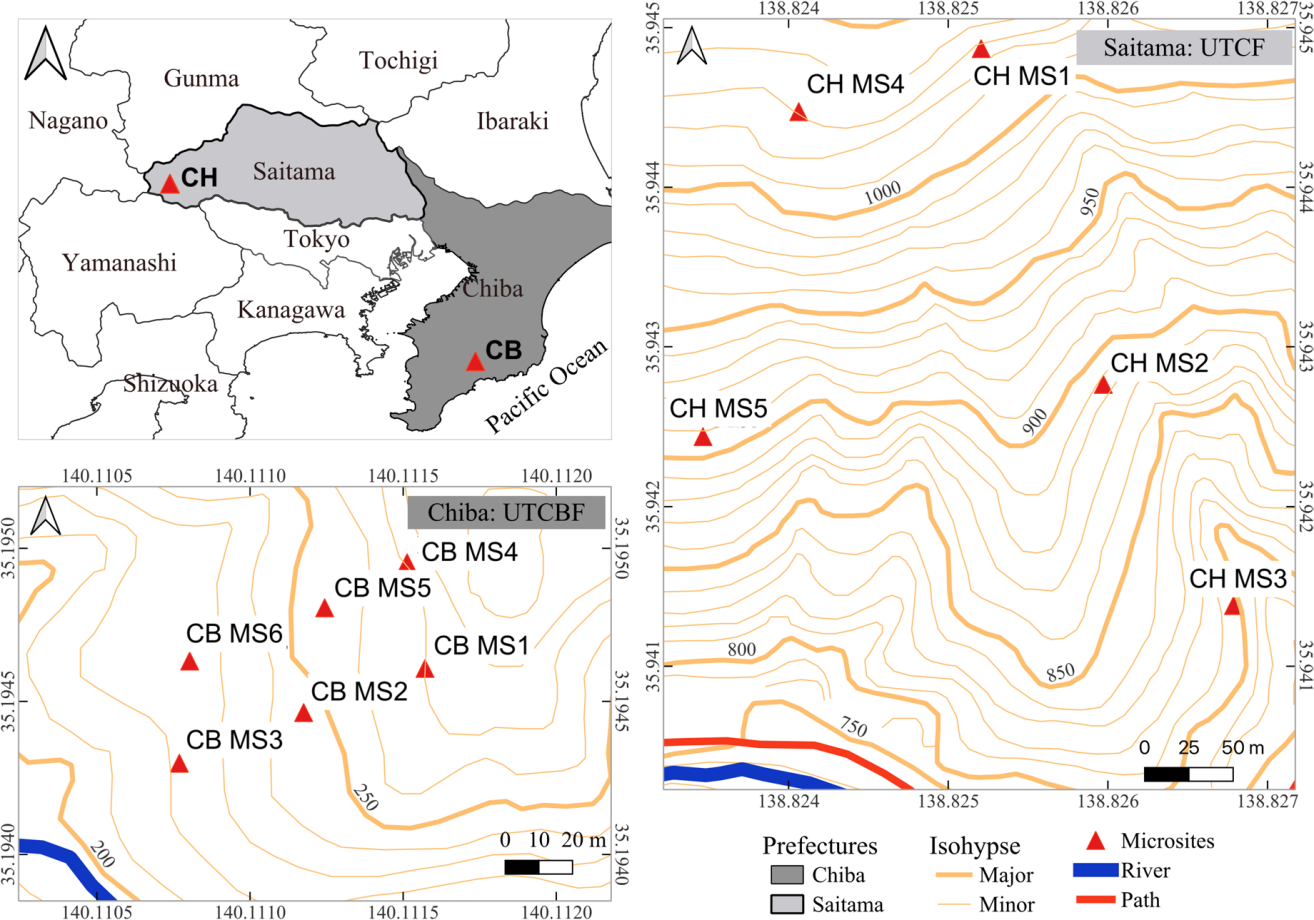
Locations of microsite plots (MS) in the study areas, illustrating the sampling design. Five individuals of *Cryptomeria japonica* were collected from each MS at the University of Tokyo Chiba Forest (UTCBF, CB) and seven from each MS at the University of Tokyo Chichibu Forest (UTCF, CH). At UTCBF, MSs 1–3 and 4–6 were planted with the cultivars “Sanbu-sugi” (SS) and “Kuro-sugi” (KS) of *Cryptomeria japonica*, respectively. Data were not available regarding the cultivars planted at UTCF

We used the sampling techniques described by Djotan et al. [33], which consist of tracking roots from the base of each targeted *C. japonica* tree, cutting them with scissors, and collecting them along with the immediately surrounding soil as a buffer. At UTCBF, we collected 30 pairs of root and soil samples from six MSs (five trees per MS) situated at various positions (upslope, middle, and downslope) on two graded topographic profiles facing southwest (Fig. 1, Table 1). Two cultivars of *C. japonica* were growing at UTCBF, namely “Kuro-sugi” (KS) and “Sanbu-sugi” (SS). At UTCF, we collected 35 pairs of root and soil samples from five MSs (seven trees per MS) located at various positions (high, medium, and low elevations) on two graded topographic profiles, one facing southeast and the other facing southwest (Fig. 1, Table 1).

**Table 1:** Summary of sampling, soil physicochemical properties, and alpha diversity of the AMF community in the roots of *Cryptomeria japonica* investigated spatially at two sites

Microsites	Trees	Longitude (°E)	Latitude (°N)	Elevation (m)	pH	TC (%)	TN (%)	C/N	TP (mg kg^−1^ soil)	No. of OTUs	Shannon
A: University of Tokyo Chiba Forest (UTCBF)											
MS1 (SS)	CB01 ~ CB05	140.1115712	35.1946080	268.4 ± 2.9 a	4.9 ± 0.6 ab	5.9 ± 3.9 a	0.4 ± 0.1 a	13.3 ± 2.3 ab	43.1 ± 18.1 b	190 ± 52 a	2.4 ± 0.5 a
MS2 (SS)	CB06 ~ CB10	140.1111750	35.1944636	246.6 ± 5.9 b	5.2 ± 0.4 a	12.8 ± 6.8 a	0.7 ± 0.2 a	16.9 ± 2.6 a	87.3 ± 27.4 b	187 ± 21 a	2.4 ± 0.3 a
MS3 (SS)	CB11 ~ CB15	140.1107692	35.1942982	215.3 ± 7.6 c	4.8 ± 0.3 ab	4.5 ± 1.5 a	0.4 ± 0.0 a	12.3 ± 1.8 b	53.8 ± 8.22 b	193 ± 52 a	2.5 ± 0.3 a
MS4 (KS)	CB26 ~ CB30	140.1115128	35.1949530	261.0 ± 5.3 a	4.6 ± 0.1 ab	6.7 ± 3.7 a	0.4 ± 0.1 a	14.4 ± 1.6 ab	71.5 ± 40.4 b	201 ± 50 a	2.3 ± 0.3 a
MS5 (KS)	CB21 ~ CB25	140.1112436	35.1948058	250.3 ± 3.1 b	4.3 ± 0.2 b	5.8 ± 0.5 a	0.4 ± 0.0 a	15.0 ± 0.7 ab	65.2 ± 7.55 b	192 ± 40 a	2.5 ± 0.2 a
MS6 (KS)	CB16 ~ CB20	140.1108030	35.1946320	218.1 ± 5.2 c	5.0 ± 0.4 ab	10.4 ± 7.1 a	0.6 ± 0.3 a	15.9 ± 3.4 ab	147.1 ± 44.6 a	189 ± 29 a	2.6 ± 0.3 a
B: University of Tokyo Chichibu Forest (UTCF)											
MS1	CH01 ~ CH07	138.82520643	35.94486857	1045.7 ± 6.6 a	5.7 ± 0.3 a	11.3 ± 1.8 b	0.7 ± 0.1 c	15.5 ± 0.5 bc	133.8 ± 67.5 ab	233 ± 43 ab	2.8 ± 0.3 a
MS2	CH08 ~ CH14	138.82597200	35.94276414	896.6 ± 11.4 c	5.1 ± 0.3 b	12.1 ± 1.4 b	0.8 ± 0.1 bc	14.7 ± 0.8 c	193.7 ± 63.8 a	177 ± 27 b	2.2 ± 0.3 b
MS3	CH15 ~ CH21	138.82678743	35.94138043	812.8 ± 9.0 d	5.3 ± 0.2 ab	11.0 ± 2.2 b	0.7 ± 0.1 c	14.7 ± 0.6 c	123.4 ± 36.0 ab	203 ± 40 ab	2.4 ± 0.5 ab
MS4	CH29 ~ CH32	138.82406358	35.94447363	1033.6 ± 6.1 a	5.5 ± 0.4 ab	18.3 ± 3.7 a	1.1 ± 0.2 a	17.0 ± 0.4 a	99.7 ± 23.6 b	244 ± 40 ab	2.4 ± 0.4 ab
MS5	CH22 ~ CH28	138.82346343	35.94243657	909.5 ± 5.4 b	5.2 ± 0.3 ab	16.3 ± 3.0 a	1.0 ± 0.1 ab	16.3 ± 0.7 ab	110.6 ± 30.4 b	254 ± 37 a	2.7 ± 0.6 ab

Values are presented as average ± standard deviation of the elevation, soil pH, TC (total carbon), TN (total nitrogen), C/N (carbon-to-nitrogen ratio), TP (total phosphorus), the number of OTUs (No. of OTUs), and Shannon index. Within a site and for the same variable (presented in columns), groups with the same letter are not significantly different (see Table S2 and Table S3). “KS” and “SS” refer to the cultivar of *Cryptomeria japonica* planted at each MS, and this information was not available for UTCF. Three samples were excluded from MS4 at UTCF after amplification of *rbc*L. *OTU*, operational taxonomic unit

### Soil Physicochemical Properties

Soil physicochemical properties were measured following the procedures described by Djotan et al. [9]. Briefly, we milled 10 g of air-dried soil samples that had been sieved through a 500-μm mesh after litter removal and digested 25–30 mg of powdered soil samples in perchloric and nitric acids. The digestion reactions were performed for 30 min and 2 h in 2 mL of nitric acid at room temperature and 120 °C, respectively, and then for 2 h in 1 mL of perchloric acid at 190 °C in an aluminum heating block. After dilution of the mixture to 5 mL, samples were incubated at 100 °C for 1 h and then cooled to room temperature. We measured TP using BIOMOL Green Reagent (Enzo Life Science, NY, USA) following the manufacturer’s instructions. TC, TN, and C/N were analyzed through dry combustion of 25–30 mg of powdered soil samples using an automatic highly sensitive NC analyzer (SUMIGRAPH NC-22F; Sumika Chemical Analysis Service, Ltd., Tokyo, Japan) that had been calibrated with the primary acetanilide standard. Soil pH was measured after the addition of 50 mL of sterilized distilled water to 20 g of air-dried soil that had been passed through a 500-μm sieve and shaking for 5 min. The mixtures were allowed to stand for 30 min, and pH was measured using a compact pH meter (LAQUAtwin-pH-33; Horiba, Kyoto, Japan).

### DNA Extraction and Amplification

We processed root samples as described by Djotan et al. [33]. Briefly, we thoroughly washed the collected basal roots under running tap water and selected all fresh first- and second-order fine root fragments. The selected root fragments were lyophilized and milled. Then, total DNA was extracted from 15–18 mg of powdered root samples using the DNeasy Plant Mini Kit (Qiagen, Germantown, MD, USA) or Extrap Soil DNA Kit Plus ver. 2 (Nippon Steel & Sumikin Eco-Tech. Co., Tokyo, Japan) according to the manufacturer’s instructions. We used Extrap Soil DNA Kit Plus ver. 2 only when the DNeasy Plant Mini Kit failed to extract DNA from a sample (two and nine samples at UTCBF and UTCF, respectively). As the choice of DNA extraction method may influence the outcome of microbial community analysis [34], using the same samples that could be DNA-extracted by both kits, we confirmed that the utilization of these two kits does not significantly affect the detected composition of AMF community in the roots of *C. japonica* (data not shown). To exclusively characterize the root AMF communities of *C. japonica*, we amplified an approximately 550-bp fragment of the *rbc*L gene according to the method of Djotan et al. [33] to check the identity of the processed root fragments and exclude inappropriate samples. For that process, amplicons of the *rbc*L gene were sequenced using the Sanger method at Macrogen Japan (Tokyo, Japan).

For nested polymerase chain reaction (PCR), we used two sets of primers, namely AML1/AML2 [35] followed by NS31/AM1 [36, 37], to amplify approximately 550 bp of the SSU rDNA and thus characterize the root AMF communities of *C. japonica*. DNA aliquots were diluted tenfold and used as templates for first-round PCR under the following cycling conditions: initial denaturation at 95 °C for 2 min; 30 cycles of 95 °C for 10 s, annealing at 58 °C for 45 s and 72 °C for 60 s; and final extension at 72 °C for 2 min. The PCR was performed in a 6 µL reaction mixture containing 1 × KAPA2G Robust HotStart ReadyMix with dye (KAPA Biosystems, Wilmington, DE, USA), 0.5 µM of each primer, and 1 µL of DNA template. The resulting PCR products were diluted 100-fold and used as templates for the second-round PCR, where the same cycling conditions were used except that the annealing step was conducted at 60 °C for 10 s. The Illumina adapters Tn5ME A and Tn5ME B were linked to the primers to allow for sample multiplexing, as described by Djotan et al. [33]. The PCR was performed in a 10 µL reaction mixture containing 2G Robust HotStart ReadyMix, 0.5 µM of each primer, and 2 µL of DNA template. The final PCR products were purified with AMPureXP beads (Beckman Coulter, Brea, CA, USA), multiplexed, and sent to Macrogen Japan for amplicon sequencing on the Illumina MiSeq platform (2 × 300 bp).

### Bioinformatics

Amplicon sequences of *rbc*L were searched using the Basic Local Alignment Search Tool (BLAST) against the National Center for Biotechnology Information (NCBI) GenBank database to verify whether the processed root samples originated from *C. japonica* trees. Then, we excluded inappropriate samples (those proven to originate from host plants other than *C. japonica*).

To characterize the AMF communities associated with the roots of *C. japonica*, we used QIIME 2 v. 2022.2.0 [38] for bioinformatic analyses unless otherwise stated. We processed the paired-read sequences using the pipeline described by Djotan et al. [8]. Briefly, reads were demultiplexed, pairs were joined, and filtering was conducted based on the q-score. Filtered sequences were used to define operational taxonomic units (OTUs) at a threshold of 97% sequence similarity. Then, we discarded rare (< 10 reads across all samples or detected in only one sample) and chimeric OTUs before BLAST searching representative OTU sequences against the NCBI GenBank, Maarj*AM* [39], and GlobalAMFungi [40] databases using the NCBI-blast-2.10.0 + program [41] to exclude non-Glomeromycotina OTUs. Taxa were assigned to OTUs based on the best matches in those databases (query cover and percent identity > 95%), and these assignments were updated following the consensus AMF classification [42].

The within-sample relative abundance of each OTU was computed and averaged for each MS. Then, the average relative abundances of OTUs among MSs were computed and used as the site average. At each site, OTUs were ranked in decreasing order of average relative abundance. An OTU was categorized as a “major OTU” and “dominant OTU” for a site when its site average relative abundance was ≥ 1% and 10%, respectively. In addition, OTUs that were dominant at a MS (average relative abundance of ≥ 10% at the MS) were also considered as dominant OTUs. Sequences of major OTUs were aligned using MAFFT v7.490 [43] and positioned on a phylogenetic tree. The tree (rooted with the outgroup *Gigaspora gigantea* MT108838) was annotated and displayed using FigTree v.1.4.4 (http://tree.bio.ed.ac.uk/software/figtree/).

### Statistical Analysis

We performed statistical analyses using R v.4.3.2 [44]. We used Euclidian distance and Ward’s criterion to cluster soil samples based on their physicochemical properties (pH, TC, TN, C/N, and TP). To estimate within-site spatial variations in soil physicochemical properties, we calculated their mean values for each MS and tested their variation among MSs with analysis of variance (ANOVA). Tukey’s honestly significant difference test at a 95% confidence level was employed when significant variation was detected in a soil physicochemical property among MSs. We performed the Shapiro–Wilk normality test and Levene’s test of homogeneity of variance to confirm normality and equality of variance prior to parametric testing. The variation among MSs of the diameter at the breast height (1.3 m aboveground, DBH) of the investigated trees was also analyzed.

We calculated the OTU richness and Shannon index (alpha diversity) values of the AMF communities in the roots of *C. japonica* using the vegan v. 2.6–4 package of R. Next, we analyzed their spatial variations as described previously. AMF community composition in each MS was ordinated using non-metric multidimensional scaling (NMDS) and tested with permutational ANOVA (PERMANOVA) to compare beta diversity among MSs. When significant spatial variation was detected among MSs at the same site, a multilevel pairwise comparison was conducted using R vegan to identify MSs with similar and dissimilar AMF communities. We also performed redundancy analysis (RDA, tested with PERMANOVA) of the relationships of AMF with soil physicochemical properties. From the RDA analysis, we extracted the AMF OTUs that showed significant contributions (*p*-value < 0.05) to the variations observed in the communities among MSs and ranked them based on their associated *R*^2^. The spatial covariations in the root AMF community, tree size (DBH), and soil physicochemical properties were analyzed with the Mantel test using Euclidean distances for pH, TC, TN, C/N, and TP, and the Bray–Curtis distance for the AMF community matrix, to calculate Spearman correlations. We also calculated Pearson’s correlation using the Hmisc R package (v. 4.7–2) to assess the associations of the AMF community’s alpha diversity with tree size.

## Results

### Soil Physicochemical Properties and DBH of Investigated Trees

All soil physicochemical properties (pH, TC, TN, C/N, and TP) differed significantly between sites, with higher values observed at UTCF (Table 1, Table S1). At UTCBF, soil pH, TP, and C/N differed significantly among MSs, while TC and TN did not (Table 1, Table S2). At UTCF, in contrast, all variables differed significantly among MSs (Table 1, Table S2). Based on the measured soil physicochemical properties, three soil clusters were identified at UTCBF (Online Resource 1). Cluster CB1 included all five samples collected at CB MS6; cluster CB2 included four of five samples collected at CB MS1, and cluster CB3 included all five samples collected at CB MS5. Samples collected at other MSs (CB MS2, CB MS3, and CB MS4) were not specific to any single cluster. At UTCF, two soil clusters were found (Online Resource 1). Cluster CH1 included all samples collected at CH MS4 and CH MS5, while cluster CH2 included all samples from CH MS2. Samples collected from other MSs (CH MS1 and CH MS3) were not specific to any single cluster. The DBH of trees was significantly different between UTCBF and UTCF (Table S1). The DBH of trees was also significantly affected by MS at both sites, but a significant difference in the average DBH among MS (Tukey’s honestly significant difference test) was observed at UTCF only (Table S2).

### Summary of Sequencing Reads

From 30 confirmed root samples of *C. japonica* collected at UTCBF, we obtained 490,650 amplicon sequences that were clustered into 553 OTUs after trimming, pair joining, and quality filtering. After the removal of chimeric, non-Glomeromycotina, and rare amplicon sequences, 436,237 validated sequences remained and were clustered into 546 OTUs, and the minimum and maximum numbers of amplicon sequences per sample were 5557 and 18,582, respectively. From UTCF, three root samples (from MS4) were excluded after analysis of *rbc*L amplicon sequences. The remaining 32 confirmed root samples of *C. japonica* yielded 503,081 amplicon sequences that were clustered into 719 OTUs after trimming, pair joining, and quality filtering. After the removal of chimeric, non-Glomeromycotina, and rare amplicon sequences, 453,992 validated sequences remained, which were clustered into 704 OTUs, and the minimum and maximum numbers of amplicon sequences per sample were 3966 and 22,565, respectively. The numbers of AMF amplicon sequences were representative of the AMF communities at UTCBF and UTCF (Online Resource 2).

### Composition of the Root AMF Communities of *C. japonica* Across MS

**Fig. 2:**
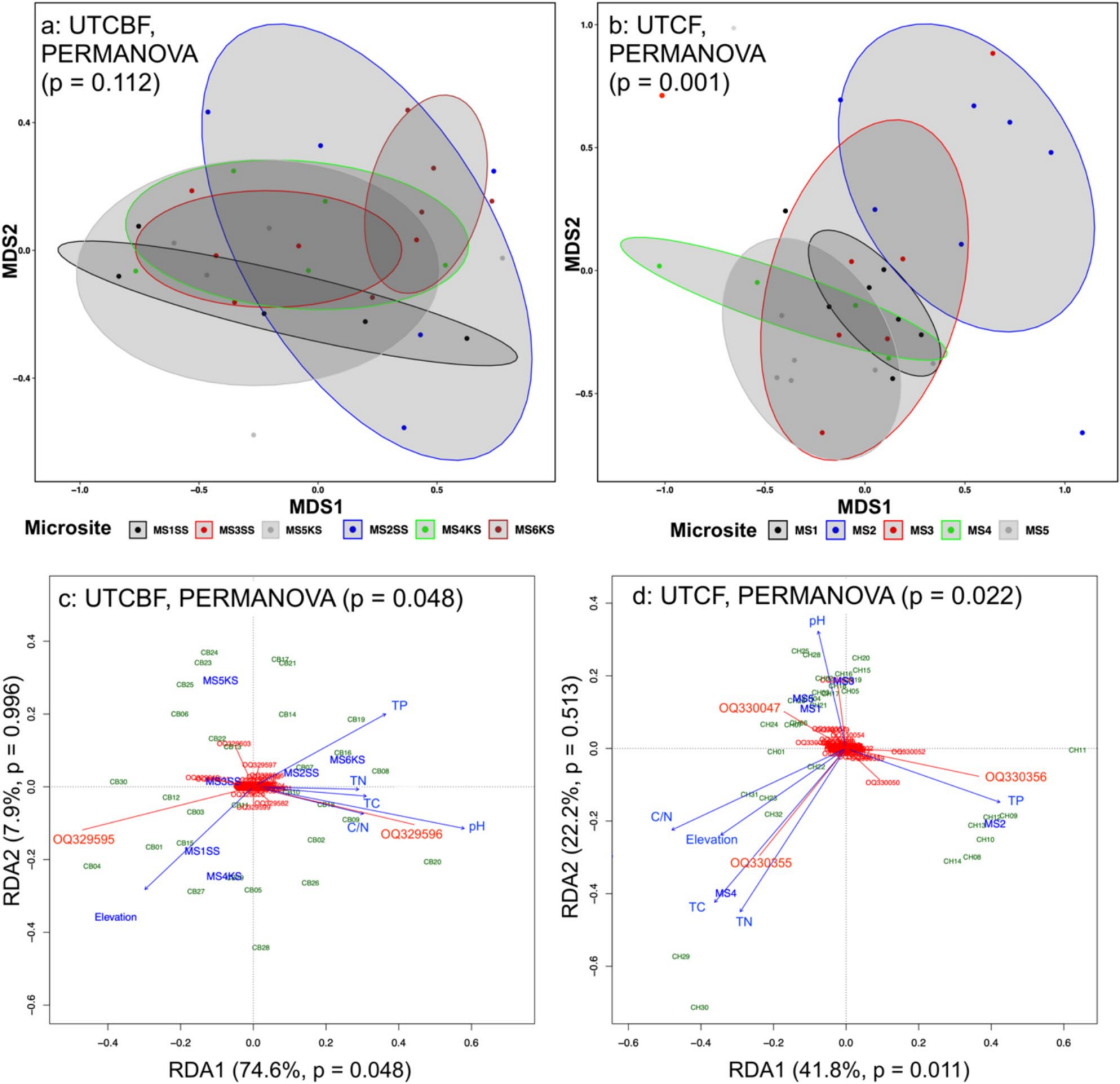
*Non-metric multidimensional scaling (NMDS) and redundancy analysis (RDA) plots of arbuscular mycorrhizal fungi (AMF) communities in the roots of* *Cryptomeria japonica* *investigated spatially at two sites*. NMDS followed by permutational analysis of variance (PERMANOVA) tests of the AMF communities in the roots of *C. japonica* among microsite plots (MS) at the University of Tokyo Chiba Forest (**a** UTCBF) and Chichibu Forest (**b** UTCF). PERMANOVA tests for RDA showing linear relationships between components at UTCBF (**c**) and UTCF (**d**). TC, total carbon; TN, total nitrogen, C/N, carbon-to-nitrogen ratio; TP, total phosphorus. In panels c and d, AMF OTUs, environmental variables, and samples are labeled in red (starting with OQ), blue, and green (starting with CB for UTCBF and CH for UTCF), respectively. KS (“Kuro-sugi”) and SS (“Sanbu-sugi”) refer to the cultivar of *Cryptomeria japonica* planted in a given MS

**Fig. 3:**
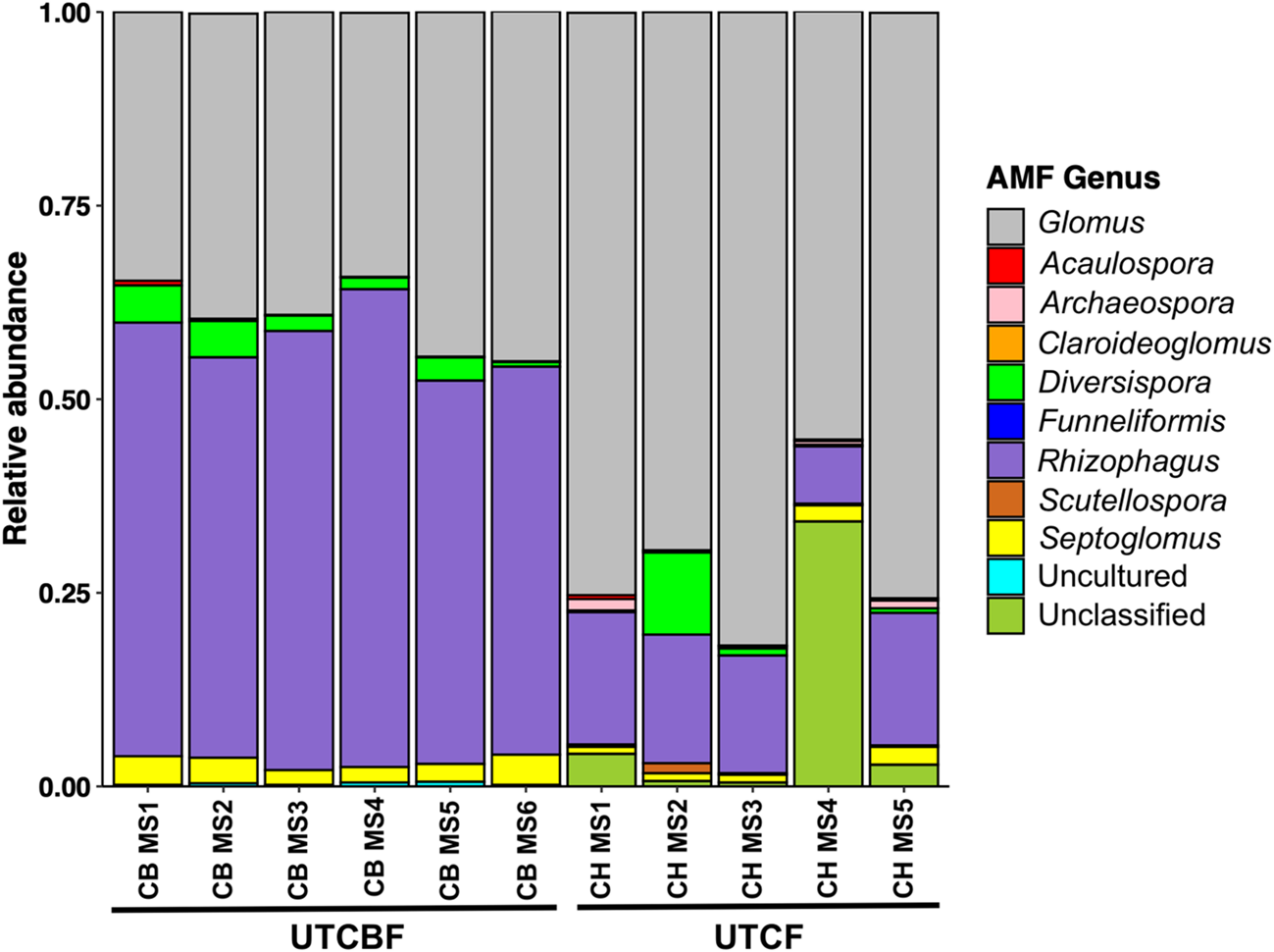
*Genus composition of arbuscular mycorrhizal fungi (AMF) communities in the roots of* *Cryptomeria japonica* *investigated spatially at two sites*. These compositions are based on the NCBI GenBank and Maarj*AM* databases. CB and CH refer to sites UTCBF and UTCF, respectively

At UTCBF, 546 OTUs were recovered from the roots of *C. japonica*. Among them, 514 belonged to Glomeraceae, 12 to Acaulosporaceae, 12 to Diversisporaceae, 2 to Archaeosporaceae, and 2 to Gigasporaceae, and 4 were unclassified (taxa not assigned due to the lack of a good match, as described previously). Among the 704 AMF OTUs recovered from the roots of *C. japonica* collected at UTCF, 669 belonged to Glomeraceae, 15 to Acaulosporaceae, 6 to Diversisporaceae, 5 to Archaeosporaceae, and 1 to Gigasporaceae, and 8 were unclassified. OTU richness and Shannon index values did not significantly differ among MSs at UTCBF but differed significantly among MSs at UTCF (Table 1, Table S3). AMF communities were significantly different among MSs at UTCF but not among MSs at UTCBF (Fig. 2, Table S4). CH MS2 had a specific AMF community that differed significantly from other MSs at UTCF (Fig. 2, Table 2). *Rhizophagus* and *Glomus* were the most abundant AMF genera in all MSs, regardless of topographic position, in the communities recovered from the roots of *C. japonica* at UTCBF and UTCF, respectively (Fig. 3, Table S5).

**Table 2:** Analysis of similarity (ANOSIM) for the arbuscular mycorrhizal fungal community of *Cryptomeria japonica* among microsite plots (MS) at the University of Tokyo Chichibu Forest (UTCF)

Microsite^a^	MS1	MS2	MS3	MS4
MS2	0.00			
MS3	0.14	0.04		
MS4	0.06	0.01	0.26	
MS5	0.07	0.00	0.38	0.40

^a ^Pairwise community similarities were not computed among MSs at the University of Tokyo Chiba Forest (UTCBF) because the AMF communities did not differ significantly among microsites. ANOSIM *p*-value < 0.05 indicates significantly different communities

### Phylogenetic Analysis and Spatial Distribution of Major OTUs Among MSs

**Fig. 4:**
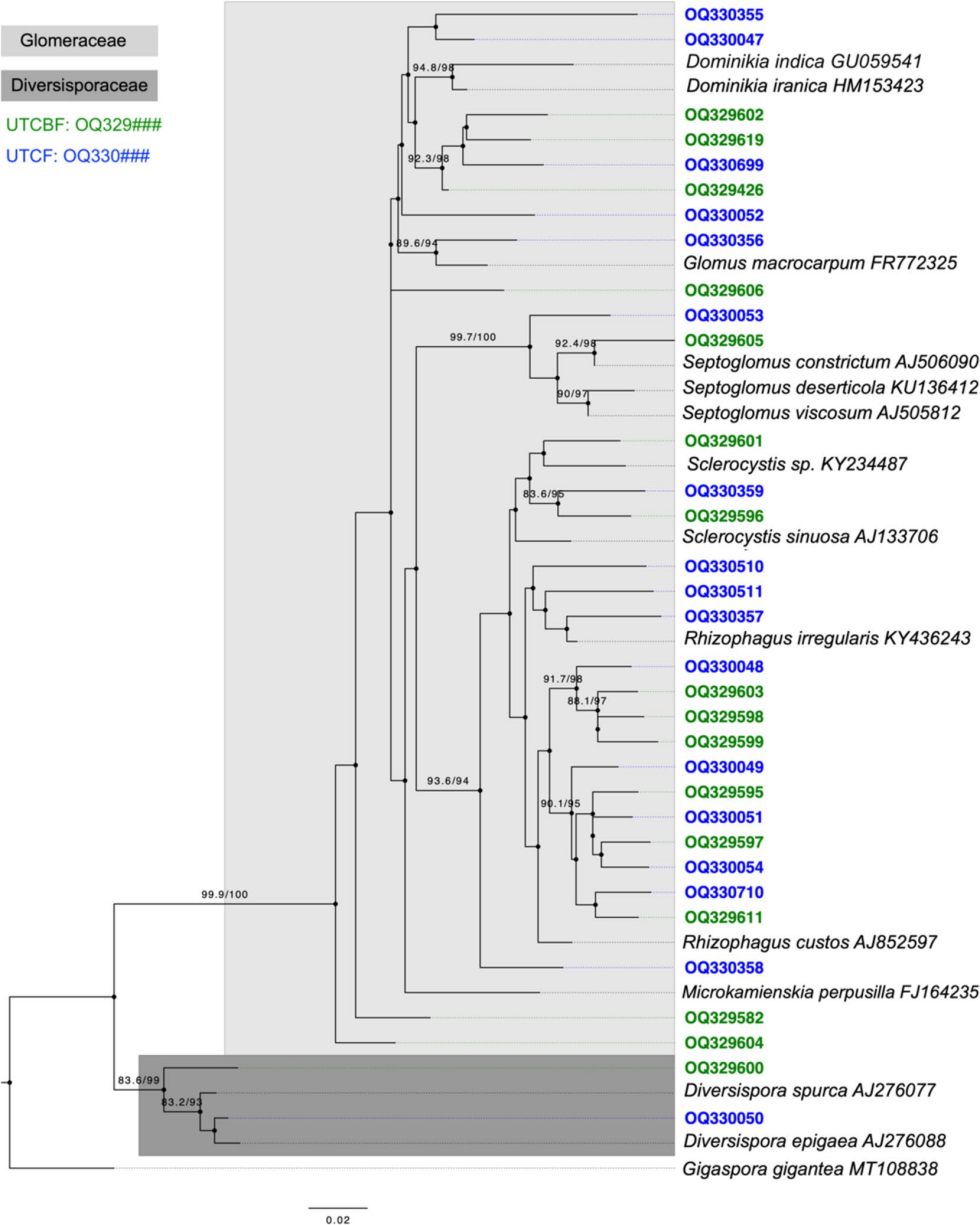
Phylogenetic tree of major operational taxonomic units (OTUs) of arbuscular mycorrhizal fungi (AMF) in the roots of *Cryptomeria japonica* investigated spatially at two sites. A maximum likelihood tree was constructed using representative sequences of major OTUs (33 nucleotide sequences) and 14 reference nucleotide sequences downloaded from the NCBI GenBank and Maarj*AM* databases. The best model and parameters were selected using the automatic model finder in IQ-TREE 2. The SH-aLRT test and ultrafast bootstrap (UFBoot) process were performed over 1000 randomizations. SH-aLRT/UFBoot are shown at nodes where SH-aLRT ≥ 80% and UFBoot ≥ 95%. Accession numbers of major OTUs (shown in green for UTCBF and blue for UTCF) and the scientific names of reference sequences followed by their accession numbers were used as labels. Aligned sequences covered 562 sites of the small subunit ribosomal DNA between primers NS31 and AM1

We detected 16 and 17 major OTUs (site average > 1%) at UTCBF and UTCF, respectively (Table 3). Three and five major OTUs were dominant at UTCBF and UTCF, respectively. The average relative abundances of major OTUs ranged from 1.1 to 24.1% among MSs. Based on the phylogenetic tree, 2 and 31 major OTUs belonged to Diversisporaceae and Glomeraceae, respectively (Fig. 4). In addition, clades of site-specific and shared major OTUs were detected. At UTCBF, three major OTUs were placed into unknown clades of Glomeraceae, while others were assigned to *Diversispora*, *Dominikia*, *Rhizophagus*, *Sclerocystis*, and *Septoglomus* (Table 3). At that site, *Sclerocystis* (OTU OQ329596) and *Rhizophagus* (OTU OQ329595) AMF were dominant with even distributions among MSs (site averages > 20%). At UTCF, two major OTUs were placed into unknown clades within Glomeraceae, and others were assigned to *Diversispora*, *Dominikia*, *Glomus, Rhizophagus*, and *Sclerocystis*. A dominant AMF of *Dominikia* (OTU OQ330047) was evenly distributed among MSs (site averages > 20%). A *Glomus* AMF (OTU OQ330356) and another *Dominikia* AMF (OTU OQ330355) showed localized dominance at CH MS2 (MS average = 28%) and CH MS4 (MS average = 30%), respectively (Table 3).

**Table 3:**
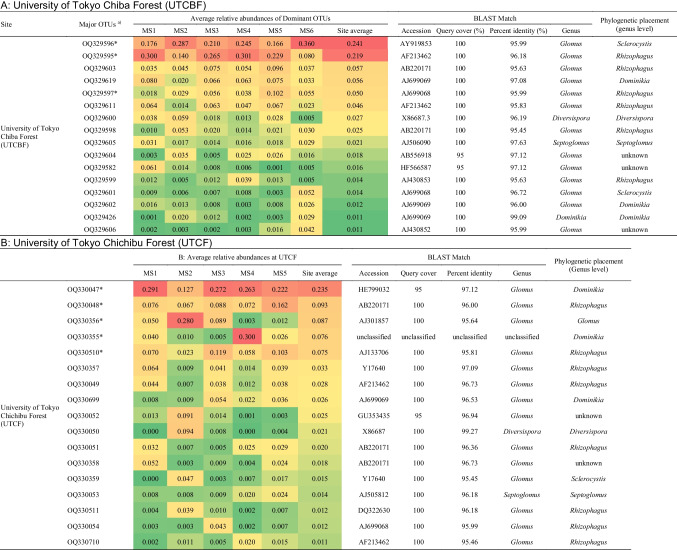
Spatial distribution of major operational taxonomic units (OTUs) of arbuscular mycorrhizal fungi (AMF) in the roots of *Cryptomeria japonica* among microsite plots (MSs)

^a)^Accession numbers of major OTUs obtained in the current study. Dominant OTUs are marked with “*.” Average relative abundances of major OTUs in the roots of trees at each MS are presented, and the average across MSs is presented as the site average (values + heatmap ranging from red for high abundance to green for low abundance). Some major OTUs formed clades, but all differed at a threshold of 100% sequence similarity (see phylogenetic tree). At UTCBF, MS 1–3 and 4–6 were planted with the *Cryptomeria japonica* cultivars “Sanbu-sugi” (SS) and “Kuro-sugi” (KS), respectively. Data were not available regarding the cultivars planted at UTCF

### Relationships of Soil Physicochemical Properties and DBH with *C. japonica* Root AMF Communities

We detected significant linear relationships between the relative abundances of the AMF in the roots of *C. japonica* and soil physicochemical properties (PERMANOVA, *F* = 1.768, *p* = 0.048) at UTCBF (Fig. 2). However, only the first axis was significant (RDA1: *F* = 14.515, *p* = 0.048), explaining 74.6% of the total variance. MS did not significantly explain the variations in the *C. japonica* root AMF community observed at UTCBF (Table 4, Table S4). Significant correlations were observed between soil pH and the relative abundances of some major AMF OTUs in the roots of *C. japonica*, particularly the dominant OTUs OQ329595 (*Rhizophagus*) and OQ329596 (*Sclerocystis*), but not for other soil physicochemical properties (Fig. 2, Table 4). The Mantel test revealed a significant positive correlation between the difference in soil pH and Bray–Curtis dissimilarity of the root AMF community (Table 5). We also detected a strong negative correlation between these two dominant AMF OTUs in the roots of *C. japonica* (Online Resource 3). While OTU OQ329595 was related to the upslope MS, characterized by low pH and TP levels, OTU OQ329596 showed the opposite trend, as it was associated with the downslope MS, characterized by high pH and TP levels (Fig. 2, Table 1). The contributions of 70 OTUs to the variation observed in the AMF community were significant (Table S6). Tree DBH significantly correlated negatively with OTU richness (*r* =  − 0.39, *p* = 0.03) but not with Shannon index (*r* =  − 0.07, *p* = 0.71). However, covariation was not significant with the AMF community composition (Table 5).

**Table 4:** Explanatory variables of variations in arbuscular mycorrhizal fungi communities in the roots of *Cryptomeria japonica*

Variables^a^	RDA1	RDA2	*r*^2^	Pr(> *r*)^b^
A: University of Tokyo Chiba Forest (UTCBF)				
TP	0.926	0.378	0.156	0.12
TC	1.000	− 0.020	0.091	0.27
TN	1.000	0.020	0.079	0.33
C/N	0.991	− 0.133	0.089	0.28
pH	0.995	− 0.100	0.322	0.01
Elevation	− 0.832	− 0.555	0.142	0.13
MS			0.274	0.08
B: University of Tokyo Chichibu Forest (UTCF)				
TP	0.986	− 0.167	0.218	0.04
TC	− 0.750	− 0.661	0.354	0.00
TN	− 0.674	− 0.739	0.314	0.00
C/N	− 0.917	− 0.398	0.345	0.00
pH	− 0.185	0.983	0.095	0.24
Elevation	− 0.864	− 0.503	0.212	0.02
MS			0.534	0.00

^a ^*TC*, total carbon; *TN*, total nitrogen, *C/N*, carbon-to-nitrogen ratio; *TP*, total phosphorus; *MS*, microsite

^b ^*p*-values < 0.05 indicate a significant correlation of the variable with the composition of the AMF community in *C. japonica* roots

**Table 5:** Mantel test results for within-site covariations in *Cryptomeria japonica* root AMF communities and soil physicochemical properties at University of Tokyo Chiba (UTCBF) and Chichibu (UTCF)

Soil properties	UTCBF		UTCF	
	Mantel statistic *r*	*p*-value^a^	Mantel statistic *r*	*p*-value^a^
pH	0.15	0.02	0.02	0.39
Total carbon (TC)	0.08	0.14	− 0.01	0.52
Total nitrogen (TN)	0.08	0.16	− 0.04	0.64
C/N	0.05	0.18	0.08	0.18
Total phosphorus (TP)	0.11	0.06	0.07	0.25
Elevation	− 0.03	0.65	− 0.04	0.69
Diameter at breast height (DBH)	− 0.03	0.63	− 0.11	0.89
All of the above	0.11	0.07	− 0.03	0.61

^a ^Significant covariation is reflected by *p*-values < 0.05

At UTCF, significant linear relationships were detected (*F* = 1.432, *p* = 0.022), and only the first axis was significant (*F* = 5.983, *p* = 0.011), explaining 41.8% of the total variance (Fig. 2). The variation in the root AMF community of *C. japonica* was significantly explained by MS and all soil physicochemical properties except pH (Table 4, Table S4). Two groups of AMF communities were detected at UTCF. AMF assemblages at CH MS1, CH MS3, CH MS4, and CH MS5 were significantly similar but were dissimilar to those at CH MS2 (Fig. 2, Table 2). Fifty-five OTUs explained a significant proportion of the variation observed in the *C. japonica* root AMF community at UTCF (Table S6). Two dominant AMF in the roots of *C. japonica*, OTUs OQ330355 (*Dominikia*) and OQ330356 (*Glomus*), showed significant positive correlations with TP, TC, TN, C/N, and elevation, but not pH (Fig. 2, Tables 3 and 4, Online Resource 3). OTU OQ330356 (*Glomus*) was associated with CH MS2, characterized by high TP levels, and was negatively correlated with OQ330047 (*Dominikia*), which was associated with CH MS1 and CH MS5 (characterized by moderate levels of TP), while OQ330355 (*Dominikia*) was associated with CH MS4, which had high levels of TC, TN, and C/N along with higher elevation (Fig. 2, Table 3). No significant correlations were observed between differences in soil physicochemical properties and the Bray–Curtis dissimilarity of the root AMF community (Table 5). Tree DBH did not significantly correlate with OTU richness (*r* =  − 0.07, *p* = 0.69) and Shannon index (*r* =  − 0.16, *p* = 0.38). Here as well, covariation was not significant with the AMF community composition (Table 5).

## Discussion

Soil physicochemical properties differed significantly among MSs at both sites (Table 1). At UTCBF, TC and TN had no significant spatial differences, whereas pH, C/N, and TP differed significantly among MSs. CB MS2 (planted with SS) and CB MS5 (planted with KS) had the highest and lowest pH, respectively. These two MSs were located in the middle of the topographic profiles, suggesting that the variation in pH among MSs was not related to the topographic position of the MS (upslope, middle, and downslope). However, at the same topographic position, pH under cultivar SS was higher than under cultivar KS, indicating that spatial variations in soil pH at UTCBF might be related to the cultivar of *C. japonica*. The SS cultivar might increase soil pH, while KS might decrease it. This result supports the previous finding of Ohta and Hiura [45] that cultivars of *C. japonica* may alter both soil pH and the accumulation of calcium in the soil. TP values were higher at CB MS6 (planted with KS) than other MSs. CB MS6 is located in the downslope portion of the profile, where the micro-topography was flat. Thus, high values of TP at CB MS6 might be due to soil erosion in the upslope area followed by drainage of phosphorus and its deposition at CB MS6. The same phenomenon of erosion-induced phosphorus loss from upslope locations and deposition at downslope locations was observed by Shi and Schulin [46].

At UTCF, all measured soil physicochemical properties (pH, TC, TN, C/N, and TP) differed significantly among MSs, which were more widely spaced than at UTCBF, suggesting that soils differed among MSs at UTCF. Based on RDA and soil cluster analysis, three AMF assemblages across two soil clusters are present at UTCF. At UTCF, CH MS1, 3, and 5 were separated from CH MS4 and CH MS2 on the RDA plot, indicating different AMF assemblages. Soils at CH MS1, 3, and 5 had high pH values; the soil at CH MS2 had high TP values and that at CH MS4 had high values of C, N, and C/N at upslope locations. However, based on the measurements taken in this study, CH MS4 and CH MS5 have similar soil properties. CH MS2 and CH MS5 are located in the middle sections of two different topographic profiles but have differing soil conditions. This finding suggests that factors other than topographic position contribute to the spatial variations in soil physicochemical properties at UTCF. Spatial variations were also observed in soil physicochemical properties among topographic positions under *Ferula sinkiangensis* [17] and *C. japonica* [47, 48] plants, with pH and C/N showing particularly strong correlations with elevation and topographic position. In these previous studies, single topographic profiles were generally investigated. In the present study, in contrast, we investigated MS distributed along two topographic profiles, allowing us to clarify that the observed variations were not solely due to the positions of the MSs on the profiles.

Soil physicochemical properties vary spatially within a given forest based on the plant community present [49, 50]. Our comparison of soil physicochemical properties among topographic positions has indicated that spatial variations may be more closely related to soil type or natural disturbances such as soil erosion than to the position along the topographic profile. Within the stand of *C. japonica* investigated at UTCBF, we unexpectedly found that soil pH differed under the two cultivars of *C. japonica*, with higher values under SS and lower values under KS trees. These findings suggest that different cultivars of *C. japonica* may have significantly different effects on soil pH. Also, previous studies have shown that the distance from the stems of trees influences soil pH via stem flow [50, 51], the same relationship might exist in *C. japonica* stands. In this study, soil pH was measured in soil surrounding the basal roots of the targeted *C. japonica* trees, located adjacent to the stems. Our observed pH values of the surrounding soil of *C. japonica* at UTCBF and UTCF were higher than that of bulk soil measured in 35 forest sites in Japan [52, 53]. These findings suggest a stratification of soil pH regarding the distance to the stem.

AMF communities in the roots of *C. japonica* were significantly similar among MSs at UTCBF but not among MSs at UTCF. Many previous studies covering different host plants have reported that soil properties determine the composition of the AMF community [13–18, 20]. In this study, we clarified the relationships of root AMF communities of *C. japonica* with soil pH, TC, TN, C/N, and TP, demonstrating within-site spatial variations. These relationships were explained primarily by specific associations of the dominant AMF taxa in the community with soil physicochemical properties. While only pH and TP differed significantly among MSs at UTCBF, all measured soil physicochemical properties differed significantly among MSs at UTCF, suggesting that soil conditions in the investigated plantations are more homogeneous at UTCBF than at UTCF. Thus, the AMF communities in the roots of *C. japonica* were more homogenous among MSs at UTCBF than at UTCF due to the spatial homogeneity in the soil conditions. Only pH was significantly correlated with the composition of the AMF community at UTCBF. The significant effect of pH on the AMF community at UTCBF was explained by significant correlations of the two dominant OTUs in the root AMF community at the site with pH. OTU OQ329596 (*Sclerocystis*) showed a positive correlation with pH and was abundant at downslope positions. In contrast, OTU OQ329595 (*Rhizophagus*) showed a negative correlation with soil pH and was abundant at upslope positions. These two dominant OTUs belonged to different genera, exhibited different correlations with pH, and were negatively correlated with each other. These OTUs might represent antagonistic AMF competing to colonize the roots of *C. japonica*. This finding supports the reported taxon-based relationships between pH and AMF [54] and the differing responses of root and soil AMF communities to pH [8, 12]. At upslope positions, OTU OQ329595 displaces OTU OQ329596, but the opposite trend was observed at downslope positions. At middle slope positions, the outcome of this competition depends on pH. For example, at CB MS2 (planted with SS), where the highest pH values were observed, OTU OQ329596 dominated the roots of *C. japonica*, while at CB MS5 (planted with KS), where the lowest pH values were observed, OTU OQ329595 dominated. These results suggest that at UTCBF, the response of the dominant AMF taxa to elevation determines the composition of the AMF community in the roots of *C. japonica* at upslope and downslope positions, whereas the response to pH is predominant at middle slope positions.

At UTCF, where all measured soil properties (pH, TC, TN, C/N, and TP) differed significantly among MSs, AMF communities in the roots of *C. japonica* also significantly differed. This result suggests that soil conditions are key determinants of the composition of the AMF community in the roots of *C. japonica* within a given site. Although pH did not explain a significant proportion of the variance observed in the total AMF community at UTCF, it played significant roles in the distributions of some major OTUs, particularly at CH MS1 and CH MS2. pH differed significantly only between CH MS1 and CH MS2, and the AMF communities at these two MSs were also significantly different. OTU OQ330047 (*Dominikia*) was highly abundant at CH MS1, which had a high pH, while OTU OQ330356 (*Glomus*) was highly abundant at CH MS2, where pH was low. These two OTUs belong to different genera, and their relative abundances were negatively correlated. Thus, these OTUs might represent antagonistic AMF competing to colonize the roots of *C. japonica*. Our findings support the evidences that AMF co-colonizing the same plant can have various ecological interactions ranging from competition to facilitation [55, 56]. The AMF corresponding to OTU OQ330047 might be competitive in more acidic soils, while that corresponding to OTU OQ330356 might be competitive in less acidic soils with high TP. Soil pH was not found to significantly affect the composition of the total AMF community at UTCF because the dominant AMF taxa at the site (OTUs OQ330355 and OQ330356) were not strongly correlated with pH. The strong positive correlation of the relative abundance of OTU OQ330356 with TP suggests that the symbiosis between *C. japonica* and this AMF may occur in soils with high TP.

The apparently competing AMF taxa exhibited different relationships with environmental factors. We found that the total AMF community in the roots of *C. japonica* at UTCBF did not significantly differ among MSs, but soil physicochemical properties consistently explained the spatial variations in dominant AMF OTUs in the community. This result supports the possibility that variations in the core AMF community (i.e., abundant and persistent AMF) can be consistently explained by soil properties, while high stochasticity might be evident in the total and satellite AMF communities of a host plant [57]. The dominant OTU OQ330355 showed significant positive correlations with TC, TN, C/N, and elevation. Thus, the AMF community in the roots of *C. japonica* was sensitive to these soil physicochemical properties. The dominant AMF OTUs in the roots of *C. japonica* collected at UTCBF formed clades with the major OTUs OQ330049, OQ330051, OQ330054, and OQ330359 detected at UTCF. However, the dominant OTUs detected at UTCF did not form any significant clades with major OTUs at UTCBF. Thus, the dominant AMF detected at UTCBF were also present as major OTUs at UTCF, whereas the dominant AMF OTUs at UTCF were not detected at UTCBF. These results suggest that the distributions at UTCBF of some major OTUs found at UTCF are limited by environmental factors such as soil conditions. Soil physicochemical properties have different impacts on the distributions of different major OTUs, explaining the differing within-site spatial variations patterns observed in the root AMF communities of *C. japonica* at UTCBF and UTCF.

Spatial variations in the AMF community have been studied at local to global scales. Communities of AMF in forest soils are reported to be spatially heterogeneous, with pairwise similarities between communities decreasing with increasing distances up to 50 m [58]. However, in accordance with findings that local environment conditions such as soil type, pH, and carbon availability determine AMF community composition at the landscape scale [14, 25, 59], we found that soil properties in planted forests of *C. japonica* affect the within-site spatial structures of its root AMF communities. Another study reported that topographic position (elevation ranging from 170 to 280 m) has a significant effect on the composition of the AMF community [22]. In that study, *Glomus* was omnipresent across the topographic gradient in plantations of *Cunninghamia lanceolata* (Chinese fir), which our findings at UTCF supported. However, our findings at UTCBF revealed the omnipresence of *Rhizophagus* among all topographic positions, suggesting site-dependent distributions of AMF taxa. In the previous study, soil properties differed among topographic positions, which may have impacted local AMF communities. Although host age might affect the AMF community [60], we did not find any significant covariation of the root AMF community composition of *C. japonica* and its DBH, except for the negative correlation of OTU richness and DBH at one of the study sites. Thus, root AMF communities are spatially homogeneous when soil physicochemical properties are homogeneous and are heterogeneous in the presence of heterogeneous soil physicochemical properties. On the other hand, it was not clear whether the spatial variation observed in the root AMF communities of *C. japonica* is associated with eventual difference in the corresponding soil AMF communities. Thus, combining data on soil physicochemical properties and soil AMF communities, like in Djotan et al. [9], would be necessary to obtain more insights into the mechanisms determining the spatial variations in root AMF communities, as well as their links to soil properties that were not covered in the present study. Within-site spatial variation in root AMF communities could also be affected by host plant species. In the plantation of *C. japonica* at UTCF, *Chloranthus serratus* (Chloranthaceae) is one of the dominant and uniformly distributed understory herb plant species [8]. Thus, in one of our studies (data presented at the International Mycological Congress IMC12), we compared the similarity of its AMF community to that of *C. japonica* and analyzed the spatial similarity of its AMF communities.

High-throughput sequencings generate huge numbers of amplicon sequences from environmental samples, and these sequences need to be clustered into taxonomic units. As discussed earlier, choice of sequence clustering approach is very important, and conclusions should consider the limitations of the chosen approach. Environmental amplicon sequences are commonly clustered into virtual taxonomic units such as VTX defined in Maarj*AM* database, OTU based on sequence similarity threshold, and ASV based on correction of a few base pairs supposed to be due to amplification and sequencing errors. The main difference between VTX, OTU, and ASV approaches is the within-unit sequence similarity. A VTX may include many OTUs, and an OTU may include many ASVs. In most recent studies of microbial communities, ASV approach, which was recommended over OTU approach (particularly for Bacteria), has gained interests [61–63]. OTU approach (particularly for AMF) itself is far from being a problem to characterizing microbial communities as lumping together similar sequences reduces the rate at which amplification or sequencing errors are misinterpreted as biological variation [62]. The ASV approaches were developed to utilize the quality of modern sequencing by including the possibility of resolving fine-scale variation [62, 64], which is not needed for AMF community studies. Also, there are growing concerns that ASVs artificially split genomes into separate clusters and OTU approach is being recommended again [65]. The ideal virtual taxonomic unit should be the one that gets us as closer as possible to species level in the system being studied. In most AMF community studies that employed the OTU approach, sequences were clustered at 97% similarity threshold, which is reasonable as AMF heterokaryosis comes with hundreds of nuclei within one continuous cytoplasm with all the nuclear genomes having an average similarity of at least 99.8% [66–70]. These evidences support our choice of methodological approach.

The AMF communities investigated here comprised 546 and 704 AMF OTUs at UTCBF and UTCF, respectively. These figures are among the highest reported among studies of AMF communities in environmental samples based on metabarcoding and Illumina MiSeq technologies. Metabarcoding of AMF in environmental samples can capture hundreds to thousands of OTUs depending on the clustering parameters [71]. However, the present study captured a very high diversity of AMF OTUs by combining the novel sampling technique and sample processing approach of Djotan et al. [33] with the conservative bioinformatic pipeline described by Djotan et al. [8] to reveal the hidden diversity, ecology, and biology of AMF communities in natural habitats. Such approaches are highly effective to observe the near-true AMF diversity of a host plant, study AMF biogeography and community ecology, and clarify the specificity of plant–AMF associations in natural habitats. Although these novel approaches are time consuming, they allow for accurate description of the AMF communities associated with tree species in natural habitats.

## Conclusion

We clarified the overlooked within-site spatial covariation of the AMF community in the roots of *C. japonica* trees with soil physicochemical properties at two environmentally different forest sites. Our hypothesis that within-site variations in soil physicochemical properties induce changes in the composition of root AMF community spatially and affect the cohabitation of dominant AMF was accepted. AMF communities in the roots of *C. japonica* within a given site are spatially heterogeneous in the presence of heterogeneous soil properties. Important soil physicochemical properties such as pH, carbon, nitrogen, and phosphorus, which are affected by the planted cultivar of *C. japonica*, determine the spatial distribution of dominant AMF taxa regardless of topographic position, leading to within-site spatial variations in the root AMF community.

## Supplementary Information

Below is the link to the electronic supplementary material.

Supplementary file1 (DOCX 1.11 MB)

## Data Availability

We deposited the sequence read archive (PRJNA898865), representative nucleotide sequences of AMF OTUs designated OQ329426–OQ329971 (SUB12643082) and OQ330017–OQ330720 (SUB12643898), and representative partial nucleotide sequence of the rbcL gene of *C. japonica* (OP832015, BankIt 2642437) in NCBI GenBank.
