# Supplementary material for: Within-Site Variations in Soil Physicochemical Properties Explained the Spatiality and Cohabitation of Arbuscular Mycorrhizal Fungi in the Roots of Cryptomeria Japonica
Source: Microb Ecol. 2024 Nov 4;87:136. doi: 10.1007/s00248-024-02449-1 (PMC11534833; doi:10.1007/s00248-024-02449-1)
Supplement: Supplementary file 1 [file 248_2024_2449_MOESM1_ESM.docx]

**Within-site variations in soil physicochemical properties explained the spatiality and cohabitation of arbuscular mycorrhizal fungi in the roots of *Cryptomeria japonica***

Akotchiffor Kevin Geoffroy Djotan ^* 1, 2, 3^, Norihisa Matsushita ^1, 4^, Kenji Fukuda ^1, 5^

**^1^**University of Tokyo, Graduate School of Agricultural and Life Sciences, Laboratory of Forest Botany, Japan

^2^ Mie University, Graduate School of Bioresources, Laboratory of Forest Mycology, Mie, Japan

ORCID: ^3^https://orcid.org/0000-0002-3726-9826; ^4^https://orcid.org/0000-0003-3281-8846, ^5^https://orcid.org/0000-0002-9980-3107

*^*^Corresponding author, E-mail:* [*geoffroydjotan@yahoo.fr*](mailto:geoffroydjotan@yahoo.fr)

## Electronic Supplementary Materials

Online Resource 1 Soil clusters based on physicochemical properties at the two study sites

a, University of Tokyo Chiba Forest (UTCBF, CB); b, University of Tokyo Chichibu Forest (UTCF, CH). Surrounding soil samples are labeled based on the trees corresponding to the root community of arbuscular mycorrhizal fungi investigated. The sample labels consist of the name of the microsite it was collected from (MSX) followed by the tree label (CBXX or CHXX)

*Online Resource 2* *Rarefaction curves of the communities of arbuscular mycorrhizal fungi in the roots of* Cryptomeria japonica *by site and microsite*

AMF, arbuscular mycorrhizal fungi; CB, University of Tokyo Chiba Forest (UTCBF); CH, University of Tokyo Chichibu Forest (UTCF); MS, Microsite

Online Resource 3 Linear regression analysis between the relative abundances of dominant operational taxonomic units (OTU) of arbuscular mycorrhizal fungi (AMF) at each site and soil physicochemical properties showing significant correlations

Sites are University of Tokyo Chiba Forest (UTCBF) and Chichibu Forest (UTCF). Soil physicochemical properties are pH (**a**); TC, total carbon (**b**); TN, total nitrogen (**c**); C/N, carbon-to-nitrogen ratio (**d**); elevation (**e**); and TP, total phosphorus (**f**)

Table S1 Comparison of environmental variables and tree diameter at breast height (DBH) between study sites

| Variables | Comparison test | Probability value |
| --- | --- | --- |
| Elevation | Kruskal-Wallis rank sum test | 0.00 |
| pH | One-way ANOVA | 0.00 |
| Total Carbon (TC) | Kruskal-Wallis rank sum test | 0.00 |
| Total Nitrogen (TN) | Kruskal-Wallis rank sum test | 0.00 |
| Total C/N | Kruskal-Wallis rank sum test | 0.05 |
| Total Phosphorus (TP) | Kruskal-Wallis rank sum test | 0.00 |
| Diameter at breast height (DBH | Kruskal-Wallis rank sum test | 0.00 |

^a)^ Significant variations reflected by p-value < 0.05

Table S2 Comparison of the diameter at breast height, elevation, and soil physicochemical properties among microsites at the study sites

A: Probabilities obtained through analysis of variance

| Variables ^i)^ | University of Tokyo Chiba Forest (UTCBF) | University of Tokyo Chichibu Forest (UTCF) |
| --- | --- | --- |
| Elevation | 0.00 | 0.00 |
| pH | 0.05 | 0.03 |
| Total carbon (TC) | 0.07 | 0.00 |
| Total nitrogen (TN) | 0.08 | 0.00 |
| Carbon to nitrogen ratio (C/N) | 0.04 | 0.00 |
| Total phosphorus (TP) | 0.00 | 0.02 |
| Diameter at breast height (DBH) | 0.04 ^ii^ | 0.01 |

B: Average values of the diameter at breast height (DBH) compared between MSs at each site

| University of Tokyo Chiba Forest (UTCBF) | |  | University of Tokyo Chichibu Forest (UTCF) | |
| --- | --- | --- | --- | --- |
| Microsites | DBH (Average ± SD, cm) ^iii)^ |  | Microsites | DBH (Average ± SD, cm) ^iii)^ |
| MS1 | 49.4 ± 2.6 a |  | MS1 | 29.0 ± 4.5 ab |
| MS2 | 53.8 ± 5.9 a |  | MS2 | 27.1 ± 1.9 b |
| MS3 | 65.2 ± 15.6 a |  | MS3 | 27.0 ± 3.9 b |
| MS4 | 52.6 ± 11.9 a |  | MS4 | 34.5 ± 3.3 a |
| MS5 | 54.2 ± 3.3 a |  | MS5 | 26.4 ± 3.3 b |
| MS6 | 65.0 ± 5.8 a |  | - | - |

^i)^ Six microsite plots (MSs) at UTCBF and five at UTCF were used for analysis of within-site spatial variations in soil physicochemical properties. ^ii)^ ANOVA showed significant effect of microsite (MS) on DBH but Tukey’s honestly significant difference test did not show any significant difference in its mean values among MSs at a 95% confidence level. ^iii)^ Within a site, groups with the same letter are not significantly different

Table S3 Probabilities obtained through analysis of variance of the number of operational taxonomic units (OTU) and Shannon index values of the root AMF communities of Cryptomeria japonica among microsite plots (MSs)

| Variables ^a)^ | University of Tokyo Chiba Forest (UTCBF) | University of Tokyo Chichibu Forest (UTCF) |
| --- | --- | --- |
| Number of OTUs | 1.00 | 0.01 |
| Shannon index | 0.38 | 0.04 |

^a)^ Six MSs at UTCBF and five at UTCF were used for analysis of within-site spatial variations in root AMF communities. Significant variations reflected by p-value < 0.05

Table S4 Permutational analysis of variance of arbuscular mycorrhizal fungal (AMF) communities in the roots of Cryptomeria japonica investigated spatially at two sites

A: Spatial variation at University of Tokyo Chiba Forest (UTCBF)

| Factor ^a)^ | Df | SumOfSqs | R^2^ | F | Pr(>F) ^b)^ |
| --- | --- | --- | --- | --- | --- |
| Microsite plot (MS) | 5 | 1.018 | 0.238 | 1.502 | 0.10 |
| Residual | 24 | 3.256 | 0.762 |  |  |
| Total | 29 | 4.274 | 1.000 |  |  |

B: Spatial variation at University of Tokyo Chichibu Forest (UTCF)

| Factor ^a)^ | Df | SumOfSqs | R^2^ | F | Pr(>F) ^b)^ |
| --- | --- | --- | --- | --- | --- |
| Microsite plot (MS) | 4 | 1.604 | 0.238 | 2.112 | 0.00 |
| Residual | 27 | 5.125 | 0.762 |  |  |
| Total | 31 | 6.729 | 1.000 |  |  |

^a)^ The effect of the cultivar of C. japonica (which was an additional factor at UTCBF) on the composition of the root AMF community was not significant. b) Significant variations reflected by p-value < 0.05

Table S5 Composition of arbuscular mycorrhizal fungal (AMF) communities in the roots of Cryptomeria japonica in various microsite plots (MSs) at the University of Tokyo Chiba (UTCBF) and Chichibu (UTCF) Forests

| AMF genus | MS at UTCBF | | | | | |  | MS at UTCF | | | | |
| --- | --- | --- | --- | --- | --- | --- | --- | --- | --- | --- | --- | --- |
|  | MS1 | MS2 | MS3 | MS4 | MS5 | MS6 |  | MS1 | MS2 | MS3 | MS4 | MS5 |
| *Acaulospora* | 0.006 | 0.003 | 0.001 | 0.001 | 0.001 | 0.001 |  | 0.005 | 0.001 | 0.001 | 0.002 | 0.003 |
| *Archaeospora* | 0.000 | 0.000 | 0.000 | 0.000 | 0.000 | 0.000 |  | 0.015 | 0.002 | 0.003 | 0.005 | 0.010 |
| *Claroideoglomus* | 0.000 | 0.000 | 0.000 | 0.000 | 0.000 | 0.000 |  | 0.000 | 0.000 | 0.000 | 0.000 | 0.000 |
| *Diversispora* | 0.048 | 0.047 | 0.020 | 0.015 | 0.030 | 0.006 |  | 0.002 | 0.106 | 0.009 | 0.002 | 0.006 |
| *Funneliformis* | 0.000 | 0.000 | 0.000 | 0.000 | 0.000 | 0.000 |  | 0.000 | 0.000 | 0.000 | 0.000 | 0.000 |
| *Glomus* | 0.347 | 0.394 | 0.391 | 0.341 | 0.445 | 0.452 |  | 0.752 | 0.695 | 0.818 | 0.553 | 0.756 |
| *Rhizophagus* | 0.560 | 0.517 | 0.567 | 0.617 | 0.495 | 0.501 |  | 0.171 | 0.166 | 0.152 | 0.074 | 0.171 |
| *Scutellospora* | 0.000 | 0.000 | 0.000 | 0.000 | 0.000 | 0.000 |  | 0.003 | 0.013 | 0.002 | 0.002 | 0.002 |
| *Septoglomus* | 0.037 | 0.033 | 0.019 | 0.020 | 0.023 | 0.039 |  | 0.009 | 0.010 | 0.010 | 0.021 | 0.023 |
| Unclassified |  |  |  |  |  |  |  | 0.042 | 0.007 | 0.005 | 0.342 | 0.028 |
| Uncultured | 0.002 | 0.004 | 0.002 | 0.005 | 0.006 | 0.002 |  |  |  |  |  |  |

Two cultivars of *C. japonica* are planted in the University of Tokyo Chiba Forest (Sanbu-sugi in MS 1–3 and, Kuro-sugi in MS 4–6), but cultivar information was not available for the University of Tokyo Chichibu Forest

Table S6 Operational taxonomic units (OTUs) making significant contributions to the variations in arbuscular mycorrhizal fungi (AMF) communities in the roots of Cryptomeria japonica among microsite plots (MSs)

A: University of Tokyo Chiba Forest (UTCBF)

| OTUs | r^2^ | Pr(>r) |
| --- | --- | --- |
| OQ329595 | 0.987 | 0.001 |
| OQ329596 | 0.969 | 0.001 |
| OQ329603 | 0.662 | 0.001 |
| OQ329619 | 0.646 | 0.001 |
| OQ329611 | 0.635 | 0.001 |
| OQ329451 | 0.616 | 0.001 |
| OQ329670 | 0.454 | 0.005 |
| OQ329899 | 0.442 | 0.015 |
| OQ329895 | 0.437 | 0.012 |
| OQ329634 | 0.422 | 0.002 |
| OQ329663 | 0.421 | 0.001 |
| OQ329698 | 0.397 | 0.002 |
| OQ329890 | 0.393 | 0.002 |
| OQ329693 | 0.393 | 0.002 |
| OQ329736 | 0.390 | 0.003 |
| OQ329858 | 0.388 | 0.006 |
| OQ329717 | 0.368 | 0.003 |
| OQ329704 | 0.366 | 0.003 |
| OQ329484 | 0.337 | 0.008 |
| OQ329669 | 0.334 | 0.008 |
| OQ329713 | 0.333 | 0.009 |
| OQ329909 | 0.331 | 0.005 |
| OQ329874 | 0.323 | 0.015 |
| OQ329633 | 0.321 | 0.011 |
| OQ329889 | 0.313 | 0.007 |
| OQ329750 | 0.312 | 0.013 |
| OQ329729 | 0.310 | 0.013 |
| OQ329538 | 0.308 | 0.015 |
| OQ329700 | 0.300 | 0.005 |
| OQ329795 | 0.300 | 0.026 |
| OQ329692 | 0.296 | 0.011 |
| OQ329902 | 0.294 | 0.019 |
| OQ329767 | 0.284 | 0.024 |
| OQ329711 | 0.282 | 0.014 |
| OQ329745 | 0.280 | 0.013 |
| OQ329805 | 0.279 | 0.025 |
| OQ329601 | 0.277 | 0.028 |
| OQ329880 | 0.276 | 0.014 |
| OQ329510 | 0.268 | 0.017 |
| OQ329605 | 0.267 | 0.012 |
| OQ329967 | 0.265 | 0.024 |
| OQ329770 | 0.264 | 0.016 |
| OQ329674 | 0.262 | 0.018 |
| OQ329940 | 0.262 | 0.027 |
| OQ329653 | 0.261 | 0.016 |
| OQ329784 | 0.260 | 0.021 |
| OQ329910 | 0.259 | 0.037 |
| OQ329661 | 0.259 | 0.022 |
| OQ329870 | 0.256 | 0.023 |
| OQ329706 | 0.255 | 0.020 |
| OQ329479 | 0.253 | 0.028 |
| OQ329606 | 0.252 | 0.022 |
| OQ329465 | 0.252 | 0.021 |
| OQ329798 | 0.241 | 0.029 |
| OQ329781 | 0.240 | 0.034 |
| OQ329632 | 0.236 | 0.033 |
| OQ329743 | 0.235 | 0.047 |
| OQ329620 | 0.227 | 0.038 |
| OQ329879 | 0.227 | 0.032 |
| OQ329680 | 0.221 | 0.037 |
| OQ329866 | 0.214 | 0.041 |
| OQ329960 | 0.213 | 0.047 |
| OQ329468 | 0.207 | 0.047 |
| OQ329641 | 0.206 | 0.030 |
| OQ329642 | 0.206 | 0.048 |
| OQ329733 | 0.206 | 0.049 |
| OQ329739 | 0.201 | 0.039 |
| OQ329648 | 0.199 | 0.028 |
| OQ329626 | 0.184 | 0.029 |
| OQ329628 | 0.178 | 0.032 |

B: University of Tokyo Chichibu Forest (UTCF)

| OTUs | r^2^ | Pr(>r) |
| --- | --- | --- |
| OQ330355 | 0.861 | 0.001 |
| OQ330356 | 0.837 | 0.001 |
| OQ330479 | 0.816 | 0.003 |
| OQ330311 | 0.755 | 0.002 |
| OQ330661 | 0.753 | 0.001 |
| OQ330531 | 0.746 | 0.001 |
| OQ330448 | 0.743 | 0.002 |
| OQ330614 | 0.696 | 0.003 |
| OQ330314 | 0.682 | 0.004 |
| OQ330649 | 0.640 | 0.001 |
| OQ330530 | 0.635 | 0.001 |
| OQ330408 | 0.630 | 0.001 |
| OQ330432 | 0.550 | 0.001 |
| OQ330551 | 0.541 | 0.002 |
| OQ330643 | 0.515 | 0.005 |
| OQ330418 | 0.507 | 0.006 |
| OQ330472 | 0.506 | 0.005 |
| OQ330573 | 0.493 | 0.004 |
| OQ330618 | 0.449 | 0.016 |
| OQ330091 | 0.436 | 0.003 |
| OQ330086 | 0.435 | 0.006 |
| OQ330202 | 0.429 | 0.009 |
| OQ330323 | 0.410 | 0.008 |
| OQ330380 | 0.405 | 0.016 |
| OQ330231 | 0.397 | 0.007 |
| OQ330568 | 0.395 | 0.006 |
| OQ330134 | 0.372 | 0.013 |
| OQ330517 | 0.368 | 0.02 |
| OQ330313 | 0.367 | 0.008 |
| OQ330245 | 0.363 | 0.007 |
| OQ330047 | 0.361 | 0.001 |
| OQ330366 | 0.350 | 0.015 |
| OQ330088 | 0.350 | 0.014 |
| OQ330228 | 0.350 | 0.019 |
| OQ330482 | 0.326 | 0.007 |
| OQ330151 | 0.323 | 0.018 |
| OQ330079 | 0.314 | 0.023 |
| OQ330164 | 0.313 | 0.006 |
| OQ330701 | 0.306 | 0.023 |
| OQ330672 | 0.301 | 0.02 |
| OQ330670 | 0.294 | 0.014 |
| OQ330226 | 0.293 | 0.024 |
| OQ330488 | 0.289 | 0.018 |
| OQ330334 | 0.271 | 0.019 |
| OQ330279 | 0.257 | 0.03 |
| OQ330172 | 0.256 | 0.025 |
| OQ330310 | 0.246 | 0.021 |
| OQ330526 | 0.240 | 0.043 |
| OQ330702 | 0.239 | 0.025 |
| OQ330510 | 0.233 | 0.048 |
| OQ330374 | 0.228 | 0.048 |
| OQ330316 | 0.219 | 0.049 |
| OQ330123 | 0.211 | 0.041 |
| OQ330087 | 0.191 | 0.044 |
| OQ330100 | 0.184 | 0.048 |

In total, 70 and 55 OTUs made significant contributions (p-value associated with RDA test less than 0.05) to the observed variations in AMF communities in the roots of C. japonica
